# Supplementary material for: Does an improved HDI trigger tourism outflows for China? New evidence from the ARDL cointegration approach
Source: PLoS One. 2025 Jul 31;20(7):e0328445. doi: 10.1371/journal.pone.0328445 (PMC12312959; doi:10.1371/journal.pone.0328445)
Supplement: Attachment — Submitted filename: revised PLOS response to reviewer comments-final-2025-6-15.docx [file pone.0328445.s002.docx]

**Review Response Sheet**

**Additional Editor Comments:**

**Editorial comment**: **The current state of literature included seems lacking. I encourage authors to explore more literature on tourism to develop a more robust theoretical framework. Particularly how factors like trade influence international trade can influence tourism.  For instance see papers:**

**1.** Çalışkan, U., Saltik, I.A., Ceylan, R., Bahar, O.: Panel cointegration analysis of relationship between international trade and tourism: Case of Turkey and silk road countries. Tour. Manag. Perspect. 31, 361–
369 (2019). https:// doi. org/ 10. 1016/j. tmp. 2019. 07. 003**.**

**2.** Fischer, C., Gil-Alana, L.A.: The nature of the relationship between international tourism and international
trade: the case of German imports of Spanish wine. Appl. Econ. 41(11), 1345–1359 (2009). https:// doi.
org/ 10. 1080/ 00036 84060 10193 49
3. AlGil-ana, L.A., Fischer, C.: International travelling and trade: Further evidence for the case of Spanish
wine based on fractional vector autoregressive specifications. Appl. Econ. 42(19), 2417–2434 (2010).
https:// doi. org/ 10. 1080/ 00036 84070 18580 83.

**4.** Azmi, S. N., Khan, T., Azmi, W., & Azhar, N. (2023). A panel cointegration analysis of linkages between international trade and tourism: case of India and South Asian Association for Regional Cooperation (SAARC) countries. *Quality & Quantity*, *57*(6), 5157-5176.

**Response:** Dear Editor, thanks for this suggestion. The discussion on the influence of international trade on tourism is expanded in the literature review section, based on the findings of the aforementioned studies.

Reviewers' comments:

Reviewer's Responses to Questions

**Comments to the Author**

1. Is the manuscript technically sound, and do the data support the conclusions?

The manuscript must describe a technically sound piece of scientific research with data that supports the conclusions. Experiments must have been conducted rigorously, with appropriate controls, replication, and sample sizes. The conclusions must be drawn appropriately based on the data presented.

Reviewer #1: Partly

Reviewer #2: Yes

2. Has the statistical analysis been performed appropriately and rigorously?

Reviewer #1: Yes

Reviewer #2: Yes

3. Have the authors made all data underlying the findings in their manuscript fully available?

The [PLOS Data policy](http://track.editorialmanager.com/CL0/http:%2F%2Fwww.plosone.org%2Fstatic%2Fpolicies.action%23sharing/1/010f01936eb8a727-828de4b4-e6e4-4d9e-991d-38fa6eb3a020-000000/AkH_xbXuaPQ6SmERsciOmgETiRXVxQAJB9vdDo41_Po=187) requires authors to make all data underlying the findings described in their manuscript fully available without restriction, with rare exceptions (please refer to the Data Availability Statement in the manuscript PDF file). The data should be provided as part of the manuscript or its supporting information or deposited to a public repository. For example, in addition to summary statistics, the data points behind means, medians and variance measures should be available. If there are restrictions on publicly sharing data—e.g. participant privacy or use of data from a third party—those must be specified.

Reviewer #1: Yes

Reviewer #2: Yes

4. Is the manuscript presented in an intelligible fashion and written in standard English?

PLOS ONE does not copyedit accepted manuscripts, so the language in submitted articles must be clear, correct, and unambiguous. Any typographical or grammatical errors should be corrected at revision, so please note any specific errors here.

Reviewer #1: Yes

Reviewer #2: Yes

**5. Review Comments to the Author**

Please use the space provided to explain your answers to the questions above. You may also include additional comments for the author, including concerns about dual publication, research ethics, or publication ethics. (Please upload your review as an attachment if it exceeds 20,000 characters)

**Reviewer #1 Comment: Dear Xie Hui et al., It was delightful to read the manuscript PONE-D-24-28502, titled “Does an Improved HDI Trigger Tourism Outflows for China? New Evidence from the ARDL Cointegration Approach.”**

**Response:** Dear Professor, thank you for taking the time to review our work. Your detailed and insightful comments have indeed helped us in enhancing the overall quality of this work. All changes are carried out using the track-changes mode of MS Word. Below is a point-by-point response to your comments.

**Comments and questions**
**Comment:** The reviewer appreciates the literature review supporting the choice of variables and the authors detailing their conceptual framework.

**Response: Thank you for your kind appreciation of our work!**

**Major comments/questions:**

**Comment: However, as they may expect, my first question is whether their data distinguished tourism as the movement of people or mobility for a short visit and migration as the same movement for a more extended period, which does not necessarily include leisure. This is because you repeatedly mentioned education leading to these outflows. Often, countries have different visa types for tourism, i.e., short stays for leisure only and for a few months usually. Education or academic visas separate because of the potential that this education might evolve into "working abroad" or "skill migration." How did you ensure that your data did not overlap some migration movements?**

**Response:** Thank you for your insightful question. We employed the "Number of departures data” provided by the World Development Indicators of the World Bank. The definition of the variable is as follows:

“International outbound tourists are the number of departures that people make from their country of usual residence to any other country for any purpose other than a remunerated activity in the country visited. The data on outbound tourists refers to the number of departures, not to the number of people travelling. Thus, a person who makes several trips from a country during a given period is counted each time as a new departure." It indicates that the data did not include those people who go abroad for work/skill migration.

**Comment: It is a no-brainer that per capita income is a deciding factor for both inbound and outbound tourism. So, when using (HDI), Are you somehow insinuating that only educated people are most likely to travel on tourism? Your education variable only mentions enrolment in higher education, not completion of any degree. So, we would need stats on completion and dropout rates to confidently use education as a key deciding factor in the discussion section.**

**Response:** Thank you for your insightful comment. First, when using HDI we are not insinuating that only educated people are most likely to travel on tourism, rather, HDI is a composite index of country's residents’ health (measured by life expectancy), education (measured by mean years of schooling and expected years of schooling), and standard of living (measured by Gross National Income (GNI) per capita). Hence, we posit that, countries with higher HDI tend to have citizens who are wealthier, healthier, and better educated—all of which tends to support higher levels of outbound tourism. Second, considering your precious comments regarding methodology section and potential multicollinearity issues we have dropped the Education variable (enrolment in higher education) from our analysis.
**Comment: Table 1: An exchange rate is a currency-based variable generated compared to another foreign currency, such as USD or EURO to CNY. Unless I missed it, the reviewer has not found any foreign currencies used to estimate the EX variable anywhere in the text. If one country in particular has been used, discussions should be limited to those two countries.**

**Response:** Thank you for your comment. We have used the real effective exchange rate index provided by the World Bank to measure our variable EX. While, as per the World Bank definition, “Real effective exchange rate is the nominal effective exchange rate (a measure of the value of a currency against a weighted average of several foreign currencies) divided by a price deflator or index of costs.” It shows that our variable EX is measured against the weighted average of several foreign currencies so we cannot mention any country in our discussion.

**Comment: GDP (Gross Domestic Product) and PPP are often highly correlated because they both measure economic activities. Since these variables are related, including both in the same model might lead to multicollinearity, making it difficult to identify each variable's effects. This correlation might signal a deeper endogeneity issue, such as reverse causality and omitted variables. Did you test for multicollinearity and endogeneity because the reviewer didn't see any of their results in the text?**

**Response:** Thank you for your helpful comment and for bringing our attention to the issues of multicollinearity and endogeneity. We have confirmed the presence of multicollinearity in our model by running the Variance Inflation Factor analysis. Therefore, we have excluded the problematic variables such as GDPPPP and population.

**Comment: Line 312: PPP is not a direct proxy for inflation. It is more of a cross-sectional tool for comparing economic well-being between countries. For example, if inflation in one country is significantly higher than in another, the first country's PPP-adjusted GDP might decline relative to the second country.**

**Response:** We have dropped this variable from our model to get unbiased outcomes.

**Comment: I found some counter-intuitive results in Table 4. How could you explain that when PPP improves, people become less likely to travel on tourism? Meanwhile, the GPD (or GDP PC) suggests otherwise.**

**Response:** We have dropped this variable from our model to get unbiased outcomes.

**Comment: Line 254 to 257: Hypothesis error
Your hypothesis does not correspond to the general framing of cointegration hypotheses. Your formulation involves testing whether individual coefficients are equal to zero, but that is not the central concern of a cointegration test. Instead, you typically test whether the variables are cointegrated (i.e., whether a long-run relationship exists). Your hypothesis specification does not align with Pesaran, Shin, and Smith's (2001) bounds-testing approach.
The correct hypothesis would be:
• Null Hypothesis (H₀): β1=β2=⋯=β6=0β1=β2=⋯=β6=0 (no cointegration).
• Alternative Hypothesis (H₁): At least one of the βi is non-zero (cointegration exists).**

**Response**: Thank you very much for this insightful suggestion. Taking heed of this recommendation, we have now adjusted the general framing of cointegration hypotheses in our revised manuscript. Kindly refer to line 351-353 in the revised manuscript.

**Comment: You provided GDP per capita in the descriptive statistics table but modeled the actual GDP. The reviewer needs clarification about which of these two variables is actually in the model. Please clarify and stick to it in the text.**

**Response:** Thanks for pointing out this thing. We have incorporated the actual GDP growth in our revised analysis.

**Comment: Could you explain why the population variable is restricted only to 15 to 64 years old? If the idea is that kids (>15) cannot travel by themselves on tourist vacations, then we can also challenge the assumption made for a 15-year-old.
The same goes for the upper-level ages. You mentioned that life expectancy has improved in China, so people now live longer and healthier lives and longevity tends to increase tourist outflows. Still, here you are again, restricting the population age to 64— This is a counterintuitive move. Understanding the ideas behind the 15—and 64-year-old age caps will be helpful.**

**Response:** Thanks for this suggestion. We have now dropped this variable from our model in this revised manuscript to get unbiased outcome.

**Comment: Lines 320 to 323: These model specifications, especially for the first and second models, are alarming for multicollinearity. GDP and income are in the same model as population and life expectancy. Please run some tests and report the results. If no issues are found, the reviewers will be happy with the results. However, it is better to check.**

**Response**: Thank you for your helpful comment and for bringing our attention to the issue of multicollinearity. We have ensured that there is no issue of multicollinearity in our model by performing the Variance Inflation Factor analysis. Therefore, we have excluded the problematic variables such as GDPPPP and population to alleviate the multicollinearity problem.

**Minor comments/questions**

**Comment: The results need to be separated from the discussion. The discussion section needs to situate the results against trends in the current literature. Is there any supporting evidence from other related studies in China and the region?**

**Response**: Thank you, we have improved and separated the discussion section from the results.

**Comment: Table 2: ADF unit root test. None of the estimates, even at the difference, have significance levels. Please follow a standard method of presenting these results and add that these are the first differences.**

**Response**: Thank you. We have improved the presentation of Table 2 by following a standard method of presenting these results and clarified that these are the first differences.

**Comment: Lines 73 to 75: In 1990, the China National Tourism Administration (CNTA) announced “Provisional measures concerning the administration of arranging Chinese citizens to three Southeast Asian countries.” Could you name the three countries mentioned? Lines 123, 148, and 154 provide similar expectations without mentioning countries.**

**Response**: Thank you. Now we have mentioned the names of the countries in the respective paragraphs. These countries are Thailand, Singapore and Malaysia. Kindly refer to line 86.

**Comment: Line 141: “Lim (49) it was…” This sentence looks odd.**

**Response:** Thank you for bringing this issue to our attention. This sentence has now been re-write. Additionally, we have performed thorough proofreading and language editing throughout the manuscript to rectify errors and enhance the quality of expression.

**Comment: Line 166: Restructure the sentence ending with North Korea.**

Response: Thanks, the sentence structure has now been improved. Kindly refer to line 214-216 in the revised manuscript.


**Comment:** Lines 193 to 198: Are these enrollments into higher education trends in China or abroad universities?

**Response:** We have removed this variable and its associated data from our research to ensure unbiased outcomes.

**Comment:** Lines 354 - 355: Please list a few countries, like the top three or five nations competing for Chinese tourists, for illustration purposes. This is important because the outflow of tourism is also based on the laws and regulations governing tourism in these host countries.

**Response:** The top five nations competing for Chinese tourists are Thailand, Japan, South Korea, France and Singapore.

**Reviewer #2: Dear Authors,**
**Comment: Congratulations for the wonderful piece of research work.
The research needs to address the following aspects:**

**Response:** Dear Professor, thank you for taking the time to review our work and appreciating our research. Your valuable comments have indeed helped us greatly in enhancing the quality of this research work. All changes have been made using the track-changes mode of Microsoft Word. Below is a point-by-point response to your comments.
**Comment: The background section need to review to make short and the sentence structure must be reviewed. For example, Line 58, 59, 63 64, 79, 107 etc.**

**Response:** Thanks for highlighting this issue. Language editing has been performed throughout the manuscript to enhance the quality of expression. Moreover, the sentences have been made as short as possible, wherever feasible, to optimise the sentence structure.

**Comment: The 3rd and 4th Paragraph in Background Section looks irrelevant. which must be revisited.**

**Response: Thanks for highlighting this issue. These paragraphs have now be adjusted in the revised manuscript.
Comment: The literature review needs more research exploration related to connecting with HDI and its components.**

**Response:** Thanks for this suggestion. Relevant studies on the association between HDI and its components, as well as outbound tourism, have been added as a paragraph under the literature review section.
**Comment: The methodology Section must be updated to verify your proposed model. If possible, verify with previous research.**

**Response:** The methodology section has been updated to justify its suitability for our proposed model in the revised manuscript. Moreover, the latest studies employing a similar econometric approach are also discussed in this section.
**Comment: The conclusion and implications section is full of evidence from previous studies where the citations are missing.**

**Response:** Thanks for highlighting this issue, relevant studies have now been cited to back the evidence discussed under this section.
**Comment: The final paragraph must be separate section: Limitations**

**Response**: Thanks for this suggestion. Limitations are now discussed under a separate section in the revised manuscript.

**Comment:** Good Luck and wish you all the best.

**Response**: Dear Prof., Thank you again for your time and efforts in helping us with valuable suggestions to improve the quality of this work. We believe that the revised manuscript is in much better shape now.
